# *CsCuAOs* and *CsAMADH1* Are Required for Putrescine-Derived γ-Aminobutyric Acid Accumulation in Tea

**DOI:** 10.3390/foods11091356

**Published:** 2022-05-06

**Authors:** Kexin Zhang, Yu Duan, Yu Cao, Yiwen Chen, Zhongwei Zou, Fang Li, Qiang Shen, Xiaowei Yang, Yuanchun Ma, Wanping Fang, Xujun Zhu

**Affiliations:** 1 College of Horticulture, Nanjing Agricultural University, Nanjing 210095, China; 2019104086@njau.edu.cn (K.Z.); 2018204034@njau.edu.cn (Y.D.); 2020104087@stu.njau.edu.cn (Y.C.); 2021104084@stu.njau.edu.cn (Y.C.); lifang@njau.edu.cn (F.L.); myc@njau.edu.cn (Y.M.); fangwp@njau.edu.cn (W.F.); 2 Department of Plant Science, University of Manitoba, 66 Dafoe Road, Winnipeg, MB R3T 2N2, Canada; zhongwei.zou@umanitoba.ca; 3 Tea Research Institute, Guizhou Provincial Academy of Agricultural Sciences, Guiyang 417100, China; shenqiang_gzu@163.com (Q.S.); yangxiaowei_gzu@163.com (X.Y.)

**Keywords:** tea, γ-aminobutyric acid, polyamine degradation, *copper-containing amine oxidase*, *aminoaldehyde dehydrogenase*

## Abstract

Polyamines are a potential source of γ-aminobutyric acid (GABA) in plants under abiotic stress. However, studies on GABA enrichment in tea mostly focus on the GABA shunt, while the correlation between polyamine degradation and GABA formation in tea is largely unknown. In this study, tea plants responded to exogenous putrescine, resulting in a significant increase in GABA content, while the glutamate level did not change. At the same time, five *copper-containing amine oxidase* (*CuAO*) and eight *aminoaldehyde dehydrogenase* (*AMADH*) genes involved in the putrescine-derived GABA pathway were identified from the Tea Plant Information Archive. Expression analysis indicated that *CsCuAO1*, *CsCuAO3* as well as *CsAMADH1* were induced to play an important function in response to exogenous putrescine. Thus, the three genes were cloned and the catalytic efficiency of soluble recombinant proteins was determined. *CsCuAOs* and *CsAMADH1* exhibited indispensable functions in the GABA production from putrescine in vitro. Subcellular localization assays indicated that *CsAMADH1* was localized in plastid, while both *CsCuAO1* and *CsCuAO3* were localized in peroxisome. In addition, the synergistic effects of *CsCuAOs* and *CsAMADH1* were investigated by a transient co-expression system in *Nicotiana benthamiana*. Our data suggest that these three genes regulate the accumulation of GABA in tea by participating in the polyamine degradation pathway and improve the content of GABA in tea to a certain extent. The results will greatly contribute to the production of GABA tea.

## 1. Introduction

γ-Aminobutyric acid (GABA) is a special four-carbon non-protein amino acid, which plays an important role involving plant growth and development [1,2]. GABA acts as an inhibitory neurotransmitter in animals, which can reduce blood pressure, relieve insomnia, depression, epilepsy and seizures [3,4,5]. The anabolism of GABA in higher plants mainly comes from the glutamate, which is catalyzed by glutamate decarboxylase (GAD), followed by GABA transaminase and succinic semialdehyde dehydrogenase before entering the tricarboxylic acid cycle [6,7]. In addition, polyamines are degraded by diamine oxidase (DAO) to form 4-aminobutyraldehyde (4-ABAL) as intermediates for GABA formation [8], which also contributes to GABA enrichment in plants besides the GABA shunt pathway. Putrescine usually undergoes a two-step reaction, including DAO or a polyamine oxidase (PAO) catalytic process, and is followed by *aminoaldehyde dehydrogenase* (*AMADH*) to form GABA in coping with external stress in plants [9,10,11].

In dicotyledonous plants, *copper-containing amine oxidase* (*CuAO*) could catalyze the putrescine into 4-ABAL, spermidine into 1,3-diaminopropane, as well as 1,3-diaminopropane into 3-aminopropionaldehyde (APAL) [12,13]. The oxidation of 4-ABAL and pyrroline is generally considered to be catalyzed by the same enzyme, NAD^+^-dependent AMADH [7], which leads to GABA biosynthesis. The preliminary evidence reported that plant AMADH activity is usually determined by substrate-dependent NADH production, which is based on crude protein extracts that convert 4-ABAL into GABA [14].

CuAO is a homodimer enzyme, not only for putrescine but also for cadaverine [15]. The molecular weight of CuAOs ranged from 70 to 90 kDa, containing a copper ion and a 2,4,5-trihydroxyphenylalanine quinone cofactor, which passes through the active site [15]. Although the overall primary sequence identities of CuAOs from different sources are usually not high (<25%) [16], most of the 33 amino acid residues near the catalytic site are completely conserved [17,18,19]. *Arabidopsis thaliana* carries ten CuAO-encoding genes, and four of them (*ATAO1* and *AtCuAO1-3*) have been identified. The gene expressions were regulated differently by development, injury and hormone or elicitor processing. The localization of CuAO protein is also different, as AtCuAO1 and TAO1 are located in exosomes, while AtCuAO2 and AtCuAO3 are peroxisomes [20,21,22]. Two genes encoding CuAO (*NtMPO1* and *NtMPO2*) in *Nicotiana Tobacum*, play important roles in the biosynthesis of pyrrolidine alkaloids [23,24]. 

Plant AMADHs exhibited the biosynthesis function of betaine aldehyde dehydrogenase, which was localized in the chloroplast [25]. Basmati or jasmine rice lacks functional 4-ABAL dehydrogenase protein and acetylates ABAL (or its cyclic pyrroline) to accumulate 2-acetylpyrroline, which is the effective ingredient of rice flavor [26]. Moreover, there are two putative AMADH genes (*AtALDH10A8* and *AtALDH10A9*) in Arabidopsis, but recombinant AtALDH10A9 can only be produced and purified in the presence of a precursor that causes the reduction of NAD^+^ [27,28]. 

To investigate the key enzymes of the polyamine degradation pathway of GABA accumulation, the gene expression of *CuAOs* and *AMADHs* in the tea plant by exogenous putrescine was determined. *CsCuAOs* and *CsAMADH1* exhibited indispensable functions in the GABA production from putrescine in vitro. Moreover, the synergistic effects of *CsCuAOs* and *CsAMADH1* were also verified by *Agrobacterium*-mediated co-expression in *Nicotiana benthamiana* leaves. Our data suggested that the *CsCuAO1*, *CsCuAO3* and *CsAMADH1* participate in the polyamine degradation pathway to form GABA, which is conducive to the accumulation of GABA in tea.

## 2. Materials and Methods

### 2.1. Plant Materials and Treatments

*Nicotiana benthamiana* and tea plants (*Camellia sinensis var. zhongcha108*) were used in this study. *N. benthamiana* plants were grown in an artificial climate incubator at 26 °C under a 16 h light (600 μmol·m^−2^·s^−1^)/8 h dark photocycle. Tea plants were grown under a cycle of 16 h light (25 °C 600 μmol·m^−2^·s^−1^)/8 h dark (20 °C). The tea plants were divided into four groups, including CK, putrescine spraying and anaerobic treatment, respectively. For the CK samples, plants were sprayed with clean water every day, and samples were taken after 0, 1, 3 and 5 days. For putrescine spraying, 5 mM putrescine spraying was applied to plants, and samples were taken after 1 day. For anaerobic, tea leaves were sealed in a bag and all the air was pumped out by vacuum, and finally, samples were taken after 8 h. The collected samples were quickly dropped into liquid nitrogen and stored in a −80 °C freezer until further analysis.

### 2.2. Determination of GABA and Glutamate Contents

A total of 0.2 g sample was ground and placed into a 10 mL centrifuge tube, 2 mL of 0.02 M HCl were then added before incubating at 4 °C for 8 h. The extracted solution was centrifuged under 4 °C at 14,000× *g* for 15 min, then 2 mL of supernatant was transferred into a new 10 mL tube. After adding 4% sulfosalicylic acid in the same volume, the extract solution was filtered by a 0.22 μm organic filter, and then the contents of GABA and glutamate were determined by an amino acid composition analyzer (Hitachi L-8900, Osaka, Japan). The contents of GABA and glutamate were obtained by calculating the peak area, which was compared to the standard solution.

### 2.3. Determination of the Putrescine Content

Putrescine content was detected by high-performance liquid chromatography (HPLC) as described by Zhu et al. [29] with a little modification. Briefly, samples were homogenized with 5% pre-cooling perchloric acid, and the homogenates were centrifuged at 12,000× *g* for 20 min under 4 °C. The supernatant was mixed with 2 M NaOH and benzoyl chloride and incubated at 37 °C for 30 min. Samples were completely mixed with diethylether and then centrifuged at 3000× *g* for 10 min at 4 °C for phase separation. The organic solvent phase was evaporated and dissolved with 0.5 mL methanol, followed by HPLC detection (C_18_ column, 15 cm × 0.39 cm × 4 μm).

### 2.4. Phylogenetic Tree Construction of Tea Plant CuAO and AMADH Gene Family

The AMADH and CuAO protein sequences of *A. thaliana*, *Populus tomentosa* and *Vitis vinifera* were derived from the plant transcription factor database, PlantTFDB (http://planttfdb.cbi.pku.edu.cn/, accessed on 15 November 2019). The rice AMADH and CuAO protein sequences were obtained from the Rice Genome Annotation Project (http://rice.plantbiology.msu.edu/index.shtml, accessed on 15 November 2019). The HMMER software was employed for specific domain searching from the tea plant genome database (http://tpia.teaplant.org/, accessed on 15 November 2019) with the default parameter E-value < 1 × 10^−5^. Pfam and PROSITE were used to verify the AMADH and CuAO domain. MEGA7.0 software, with default parameters, was used for constructing a neighbor-junction (NJ) phylogenetic tree. The prediction of amino acid sequence features and the motifs of the amino acid sequence was conducted by the ProtParam tool (https://web.expasy.org/protparam/, accessed on 15 November 2019) and MEME (http://meme-suite.org/tools/meme, accessed on 15 November 2019), respectively.

### 2.5. Gene Expression Analysis

Total RNA was isolated and then reverse transcribed using a Plant Total RNA isolation Kit Plus (Foregene biotech Co. Ltd., Chengdu, China) and a HiScript II Q RT SuperMix (Vazyme biotech Co. Ltd., Nanjing, China) according to the manufacturer’s instruction, respectively. The quantitative real-time PCR assays (Bio-Rad, Houston, TX, America) were performed in the public platform of the laboratory of the College of Horticulture, Nanjing Agricultural University. The reagent used in the qRT-PCR experiment is ChamQ Universal SYBR qPCR Master Mix (Vazyme biotech Co. Ltd., Nanjing, China). The relative gene expressions were calculated using the 2^−ΔΔCT^ method, in which *Csβ-actin* was selected as the internal control. The primer pairs used in this study were listed in Table S1. 

### 2.6. Purification of CsCuAO1, CsCuAO3 and CsAMADH1 In Vitro

*CsCuAO1*, *CsCuAO3* and *CsAMADH1* from tea leaves (*Camellia sinensis var. zhongcha108*) were cloned by specific primer pairs, and are listed in Table S1. The followed protocol was used: denature for 2 min at 94 °C; followed 35 cycles of the sequence: 30 s at 94 °C, 30 s at 56 °C, and 1 min at 68 °C for annealing. The PCR product was purified, and restriction enzymes were cut and ligated into a pGEX-4T-1 vector (CWBio Co. Ltd., Shanghai, China).

### 2.7. CsCuAOs and CsAMADHs Activities Assay

*CsCuAOs* activities were determined as described by Tipping and McPherson [30], with a little modification. Samples were homogenized in 0.1 M potassium phosphate buffer (pH 6.5) and then centrifuged at 15,000× *g* for 15 min at 4 °C. The supernatant was transferred to a new tube for enzyme reaction, 4-aminoantipyrine/N, N-dimethylaniline chromogenic solution, and 0.1 mL horseradish peroxidase were added. The reaction was initiated by adding 0.02 M putrescine solution, then detected the absorbance at 555 nm by a spectrophotometer (Hitachi UH4150, Osaka, Japan). 

For *CsAMADH1* activity, the crude enzymes were extracted according to the method described by Petrivalský et al. [31]. The samples were homogenized using 0.1 M potassium phosphate buffer (pH 8.0, including 0.005 M DTT, 0. 1 mM EDTA and 10% sucrose). The enzyme extracts were transferred to a new tube, followed by adding 0.1 M potassium phosphate buffer (pH 8.0), 0.001 M NAD^+^. The reaction was initiated by adding 0.1 mM of 4-ABAL, then the absorbance at 340 nm by a spectrophotometer was detected. 

To investigate the K*_m_* value for 4-ABAL and GABA formation, a series of precursors from 0.1 mM to 100 mM were set for the enzyme reaction solution. 

### 2.8. Subcellular Localization Analysis of CsCuAO1, CsCuAO3 and CsAMADH1

The recombinant proteins of *CsCuAO1*-GFP, *CsCuAO3*-GFP and CsAMADH-GFP transformed into *Agrobacterium tumefaciens* strain GV3101 cells, the vector pBI121-GFP was used as a control [32]. The transient expressed *N. benthamiana* plants were put in a plant incubator in the dark overnight and followed a 16 h light (600 μmol·m^−2^·s^−1^)/8 h dark photocycle for two days. Finally, *N. benthamiana* leaves were detected using an LSM800 ultra high-resolution confocal microscopy imaging system (Zeiss Co., Oberkochen, Germany).

### 2.9. Statistical Analysis

The data were calculated using Excel (Microsoft Office 2016, Seattle, WA, USA) from three replicates. Both ANOVA and Duncan’s test were employed for significance analysis. 

## 3. Results

### 3.1. Changes in GABA, Glutamate, Putrescine Contents under Different Treatments

**Figure 1:**
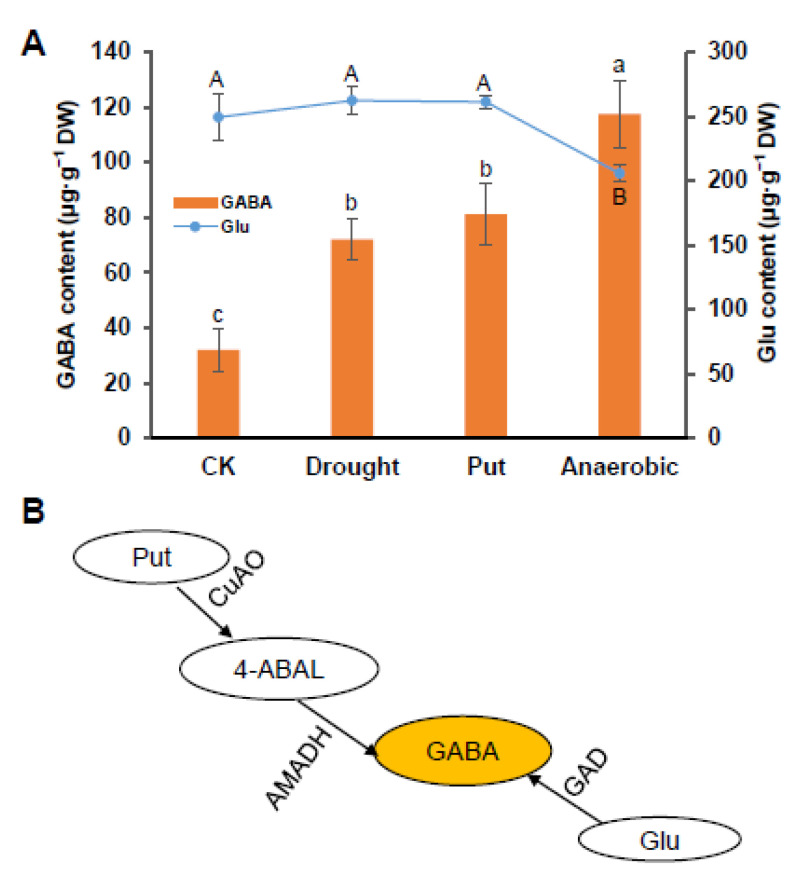
The accumulation profiles of GABA under various different treatments. (**A**) The quantitative analysis of GABA and Glu under drought, anaerobic, Put spraying and mechanical damage. Data represent the mean value ± standard deviation; means with different letters are significantly different from each other (*p* ≤ 0.05). Capital letters and lowercase letters represent Glu and GABA, respectively. (**B**) A schematic drawing of the GABA biosynthesis pathway. Glu, glutamate; Put, putrescine.

The GABA levels in leaves increased significantly after putrescine spraying and anaerobic treatment, compared with the control tea plants (Figure 1A). Interestingly, the content of glutamate exhibited different trends under both two treatments. The glutamate contents had no significant change compared with the control group under putrescine spraying treatment. However, for anaerobic treatment, the glutamate content decreased significantly. There are two pathways in GABA synthesis: one is the GABA shunt regulated by GADs, and the other is the polyamine degradation pathway, which is divided into two steps, catalyzed by CuAOs and AMADHs, respectively (Figure 1B).

### 3.2. Phylogenetic Analysis of Tea Plant CuAO and AMADH Gene Family and Gene Expression 

**Figure 2:**
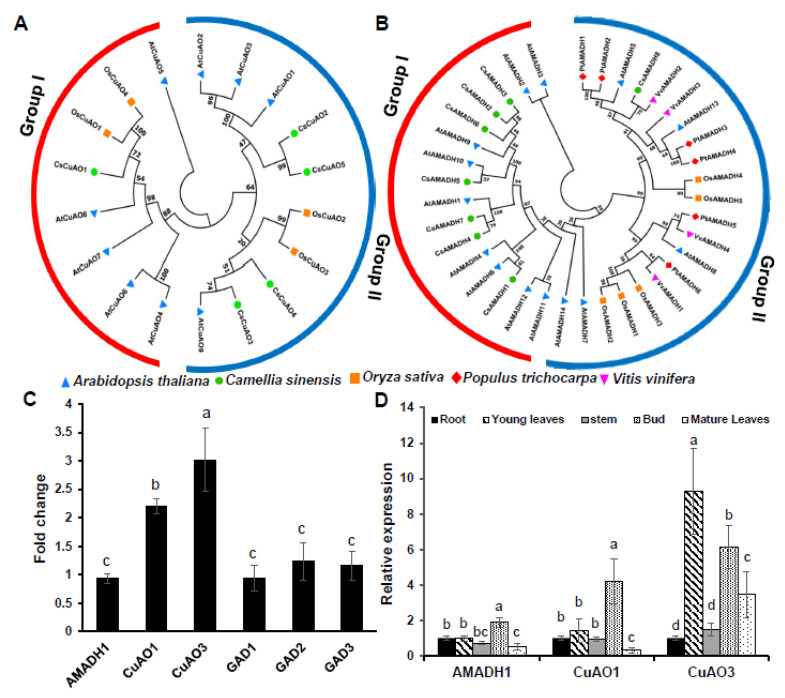
The expression profiles of CsCuAO and CsAMADH family members. Phylogenetic tree showing clustering of CsCuAO (**A**) and CsAMADH (**B**) family members from *C. sinensis* and other plant species. (**C**) The fold change of three genes and GADs expression by putrescine spraying treatment. The lowercase letters present over the column indicate significant differences by treatment (*p* < 0.05) (**D**) The expression patterns of three genes in various organs. The lowercase letters present over the column indicate significant differences among different organs (*p* < 0.05).

Five *CuAO* and eight *AMADH* genes were strictly identified from the Tea Plant Information Archive. Phylogenetic tree analysis showed that *CsCuAOs*’ family can be divided into two groups (Figure 2A) and the *CsAMADHs* family also has two groups (Figure 2B). The detailed sequences and conserved motifs of *CsCuAOs* and *CsAMADHs* were analyzed (Figure S1).

Interestingly, the expression of *CsAMADH1* and *CsGADs* did not increase obviously by putrescine application, but the expression of *CsCuAO1* and *CsCuAO3* increased significantly, which the fold change value of *CsCuAO1* and *CsCuAO3* was 2.2 and 3, respectively (Figure 2C). Furthermore, there were no apparent changes in the expression of *CsGADs* under the putrescine spaying (Figure 2C). For tissue-specific analysis, the expression of *CsAMADH1* and *CsCuAO1* showed the highest expression in buds and the lowest in mature leaves, while the expression of *CsCuAO3* was highly expressed in young leaves, buds and mature leaves, and the expression pattern showed the highest in young leaves while the lowest in roots in the tea plant (Figure 2D).

### 3.3. Purification and Enzyme Kinetics of CsCuAO1, CsCuAO3 and CsAMADH1

**Figure 3:**
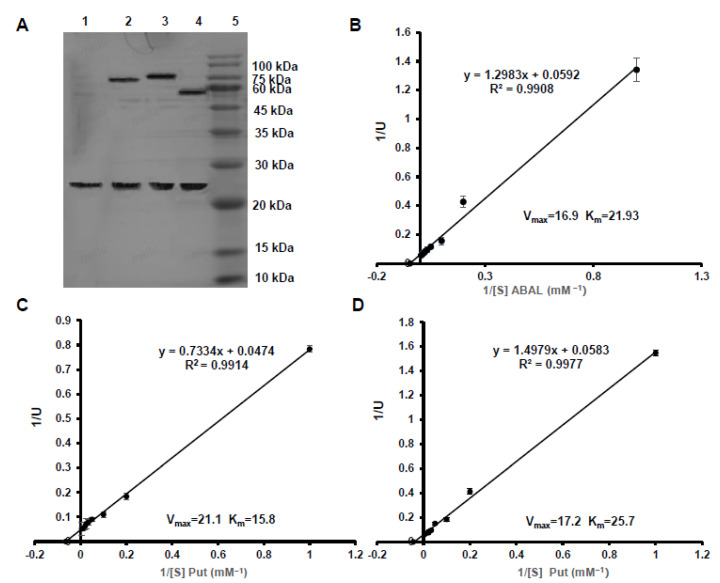
Properties of *CsCuAO1*, *CsCuAO3* and *CsAMADH1*. (**A**) The sodium dodecyl sulfate polyacrylamide gel electrophoresis analysis of the recombinant GST-fusion protein. Lane 1: empty vector; Lane 2: *CsCuAO3*; Lane 3: *CsCuAO1*; Lane 4: *CsAMADH1*; Lane 5: protein molecular weight marker. Lineweaver–Burk plot of *CsCuAO1* (**B**), *CsCuAO3* (**C**) and *CsAMADH1* (**D**).

The *CsCuAO1*, *CsCuAO3* and *CsAMADH1* proteins with the GST tag were isolated and purified, and the sizes of these proteins were 79.6, 74.8 and 55.0 kDa, respectively (Figure 3A). Lineweaver–Burk plots (Figure 3B–D), calculated from the linear formula (1/U against 1/[S]), revealed the V*_max_* values for the converting of *CsCuAO1* and CsCuAO2 to putrescine were 16.9 and 21.1 μmol·mg^−1^·min^−1^, respectively. The V*_max_* values for the conversion of *CsAMADH1* to 4-ABAL was 17.2 μmol·mg^−1^·min^−1^. Moreover, the K*_m_* values of *CsCuAO1*, *CsCuAO3* and *CsAMADH1* were 21.9, 15.8 and 25.7 Mm, which indicated that the substrate-binding affinity of the three enzymes was definitely discrepant. 

**Figure 4:**
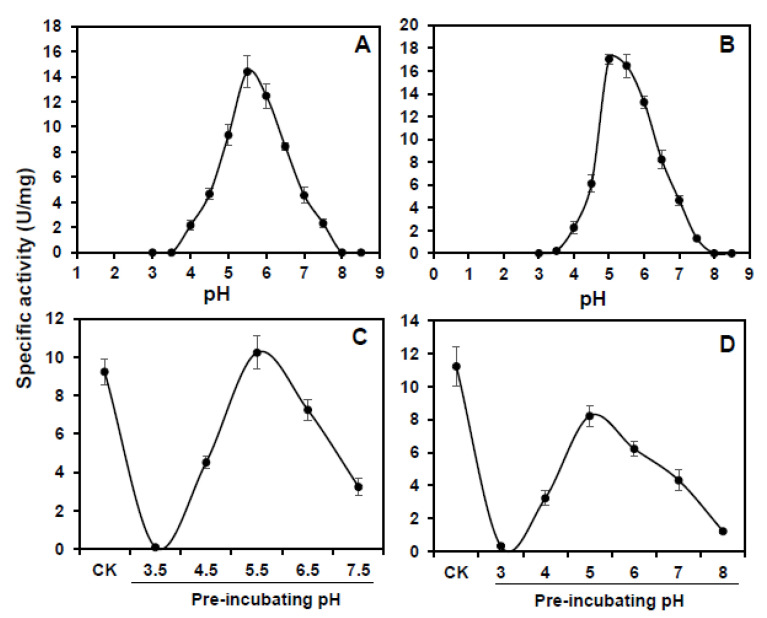
Activity and stability of *CsCuAOs* under different pH conditions. Activity of *CsCuAO1* (**A**) and *CsCuAO3* (**B**) was determined between pH = 3 to pH = 8. Stability of *CsCuAO1* (**C**) and *CsCuAO3* (**D**) was detected by incubating with a series of pH buffers for 12 h at 4 °C and then assayed the enzyme activity at pH = 5.5 and pH = 5.0, respectively. CK, the enzyme stored at −80 °C, was used as the control.

**Figure 5:**
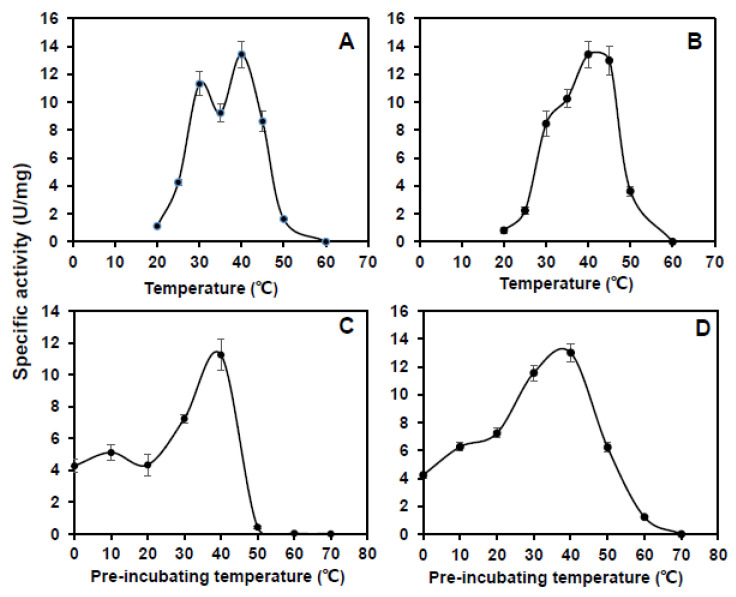
Activity and stability of *CsCuAOs* under temperature. Activity of *CsCuAO1* (**A**) and *CsCuAO3* (**B**) was determined between 20–60 °C. Stability of *CsCuAO1* (**C**) and *CsCuAO3* (**D**) was detected by incubating at a series of temperatures for 30 min, and then assayed the enzyme at 40 °C, respectively.

The maximum enzyme activity of *CsCuAO1* and *CsCuAO3* was detected at pH 5.5 (Figure 4A,B). The performance of pH stability analysis revealed that *CsCuAO1* activity was restored after a 12 h treatment under pH 4.5–7.5, while the enzyme activity disappeared under treatment at pH 3.5. *CsCuAO3* activity was gradually restored at pH 4.0–8.0 and was eliminated at pH 3.0, and the pH stability of *CsCuAO3* was more stable than *CsCuAO1* (Figure 4C,D). The most active temperature for both *CsCuAO1* and *CsCuAO3* was 40 °C (Figure 5A,B). Additionally, the detection of thermal stability revealed that pre-incubation temperatures of more than 40 °C decreased the activity of both *CsCuAOs* (Figure 5C,D), especially *CsCuAO1*, which is almost completely inactivated when the pre-incubation temperature reaches 50 °C. These results implied that the enzyme activity of *CsCuAO3* was more stable than *CsCuAO1*, and the temperature had a greater effect than that of pH.

### 3.4. Assays of CsCuAO1, CsCuAO3 and CsAMADH1 Enzyme Activity on GABA Production In Vitro

**Figure 6:**
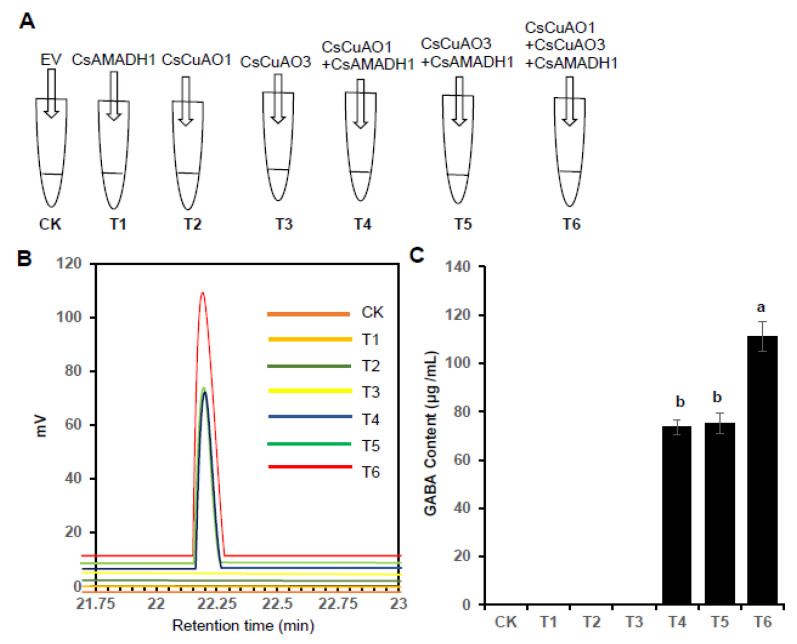
Assays of *CsCuAO1*, *CsCuAO3* and *CsAMADH1* enzyme activity on GABA production in vitro. (**A**) A simplified scheme showing the enzyme reaction. (**B**) Representative amino acid chromatograms for enzymatic reactions with substrate. (**C**) GABA accumulated in different enzyme reactions. The lowercase letters present over the columns indicate significant differences among different treatments (*p* < 0.05).

In order to identify whether *CsCuAOs* and *CsAMADHs* have synergistic effects, we carried out validation experiments on the three proteins in vitro and treated them with seven groups (CK and T1–T6) with putrescine as the substrate (Figure 6A). The GABA content, as the reaction product, was determined by an amino acid analyzer (Figure 6B). What can be obviously seen, was that there is no GABA production in either the CK group or the T1, T2 and T3 groups. However, GABA was produced in the groups of T4, T5, T6, and the GABA content of T6 was much higher than T4 and T5 (Figure 6C). These results indicated that *CsCuAOs* and *CsAMADH1* were indispensable for the GABA production from putrescine in vitro.

### 3.5. Transient Transformation Expression in Nicotiana Benthamiana

**Figure 7:**
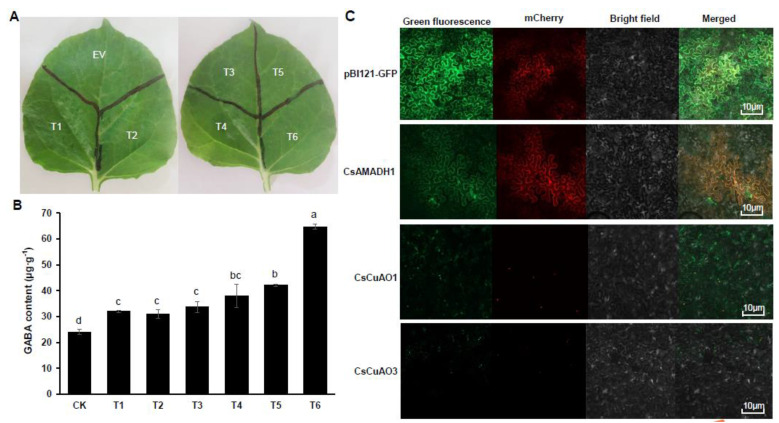
Transient assays of *CsCuAO1*, *CsCuAO3* and *CsAMADH1* in *N. benthamiana* leaves. (**A**) The phenotypes of *N. benthamiana* leaves by Agrobacteria infiltration harbor the respective plasmids after 1 day. (**B**) GABA accumulated in leaves at 3 days after agro-infiltrations. The lowercase letters present over the columns indicate significant differences among different treatments (*p* < 0.05). (**C**) The subcellular localization of GFP fusion proteins of *CsCuAO1*, *CsCuAO3* and *CsAMADH1*. The vector pBI121-GFP was used as control, and the mcherry was used as plastid and peroxisome, respectively. Scale bars were 10 μM.

Based on the in vitro results, we further carried out the validation of three genes in vivo—the *Agrobacterium*-mediated *Nicotiana Benthamiana* transient assay (Figure 7A), and GABA in leaves was detected after agro-infiltration for three days. The results suggested that the GABA level in *N. Benthamiana* leaves increased by agro-infiltration with a single gene, while the content of GABA in leaves could increase more by agro-infiltration with two-step genes simultaneously (Figure 7B). In fact, the GFP fusion subcellular localization analysis indicated that *CsAMADH1* was localized in plastid, while both *CsCuAO1* and *CsCuAO3* were localized in peroxisome (Figure 7C).

## 4. Discussion

GABA is considered to be the major amino acid synthesized under anaerobic conditions, which are formed by glutamate decarboxylation based on GAD function [33,34]. The GABA level was increased significantly, and the content of glutamate decreased, indicating that the increased GABA under hypoxia is dominated by the GABA shunt pathway (Figure 1A) [35,36]. However, GABA content increased but glutamate did not change under the putrescine spraying treatment, which indicates that the GABA shunt did not respond to the induction of exogenous putrescine, and the increase in the GABA content is through polyamine degradation. For gene expression patterns, the putrescine degradation pathway genes, including *CsCuAO1*, *CsCuAO3* and *CsAMADH1* could respond to exogenous putrescine (Figure 2D). However, in the GABA shut pathway genes, the expression of *CsGADs* was only induced under anaerobic stress (Figure S2). Subcellular localization results indicated that *CsAMADH1* was localized in plastid, and both *CsCuAO1* and *CsCuAO3* were localized in peroxisome (Figure 5C). In Arabidopsis, the localization of CuAOs protein was different, which AtCuAO1 was localized in ectoplast, while AtCuAO2 and AtCuAO3 were found in peroxidases [16,37,38]. Taken together, *CsCuAO1*, *CsCuAO3* and *CsAMADH1* could respond to the induction of exogenous putrescine and regulated the polyamine degradation pathway to increase the content of GABA in tea. Under anaerobic conditions, the significant increase in GABA content is dominated by the GABA shunt and its key genes *CsGADs*.

Our previous studies have revealed that 1/4 of GABA produced in tea leaves under anoxia comes from polyamine (predominantly putrescine) degradation [35]. In order to further study the functions and characteristics of these three genes and their enzyme synthesis, we purified their recombinant proteins and carried out a series of function verification experiments in vivo and in vitro. In the present study, we found that the substrate-binding affinity of *CsCuAO3* was greater than that of *CsCuAO1*, as the K*_m_* value of *CsCuAO3* was lower than *CsCuAO1* (Figure 3). In addition, *CsCuAO1*, *CsCuAO3* and *CsAMADH1* exhibited very high enzyme activity in vitro and in vivo played for GABA formation (Figure 6 and Figure 7). It is reported that polyamines are degraded by DAO to form 4-ABAL intermediates, followed by AMADH catalyzation, which is another way for GABA enrichment in plants [39]. DAO takes putrescine as substrate in cowpea seedlings, in which K*_m_* and V_max_ are 0.15 mM and 0.065 mol·min^−1^, respectively, while the oxidation activity of spermidine and spermine is only 16% and 38% of putrescine (Petrivalský et al., 2007). In this study, we revealed that the synergistic effects of *CsCuAOs* and *CsAMADH1* were shown by a transient co-expression system (Figure 7). Therefore, the present study demonstrated that these three genes are involved in GABA production through polyamine degradation in the tea plant.

It appears that there may exist a totally different pathway between the accumulation of GABA levels and the response to putrescine spraying treatment or hypoxia stress. Thus, we studied the accumulation of GABA in tea from the perspective of the polyamine degradation pathway and clarified its mechanism to a certain extent. The results showed that the *CsCuAOs* and *CsAMADH1* were key genes in the polyamine degradation pathway. However, GADs were considered the dominant genes for regulating GABA formation under anaerobic conditions, in which the expression levels of *CsGAD2* and *CsGAD3* increased significantly, 3.5- and 2.4-fold, respectively (Mei et al., 2016; Figure S2).

## 5. Conclusions

The functions of three key enzymes involved in GABA production from the polyamine degradation pathway were analyzed in this study. The three genes employed by the putrescine-derived GABA accumulation in the tea plant were firstly reported. Our data showed that *CsCuAO1*, *CsCuAO3* and *CsAMADH1* were the key genes involved in GABA production in the tea polyamine degradation pathway and it was driven by the combined synthesis of *CsCuAO1*, *CsCuAO3* and *CsAMADH1*, which will have a great contribution to the production of GABA Tea. 

## Data Availability

All data are available in the manuscript or the Supplemental Materials.

## Supplementary Materials

The following supporting information can be downloaded at: https://www.mdpi.com/article/10.3390/foods11091356/s1, Figure S1: Schematic drawing of *CsAMADH* and *CsCuAO* family members; Figure S2: CsGADs were induced under hypoxia treatment, but not *CsCuAOs* and *CsAMADH1*. The lowercase letters present over the columns indicate significant differences among different genes (*p* < 0.05). Table S1: Primers used in this study.
