# Supplementary material for: CsCuAOs and CsAMADH1 Are Required for Putrescine-Derived γ-Aminobutyric Acid Accumulation in Tea
Source: Foods. 2022 May 6;11(9):1356. doi: 10.3390/foods11091356 (PMC9100525; doi:10.3390/foods11091356)
Supplement: Supplementary file 1 [file foods-11-01356-s001.zip › Supplementary Figures-20220327.pdf]

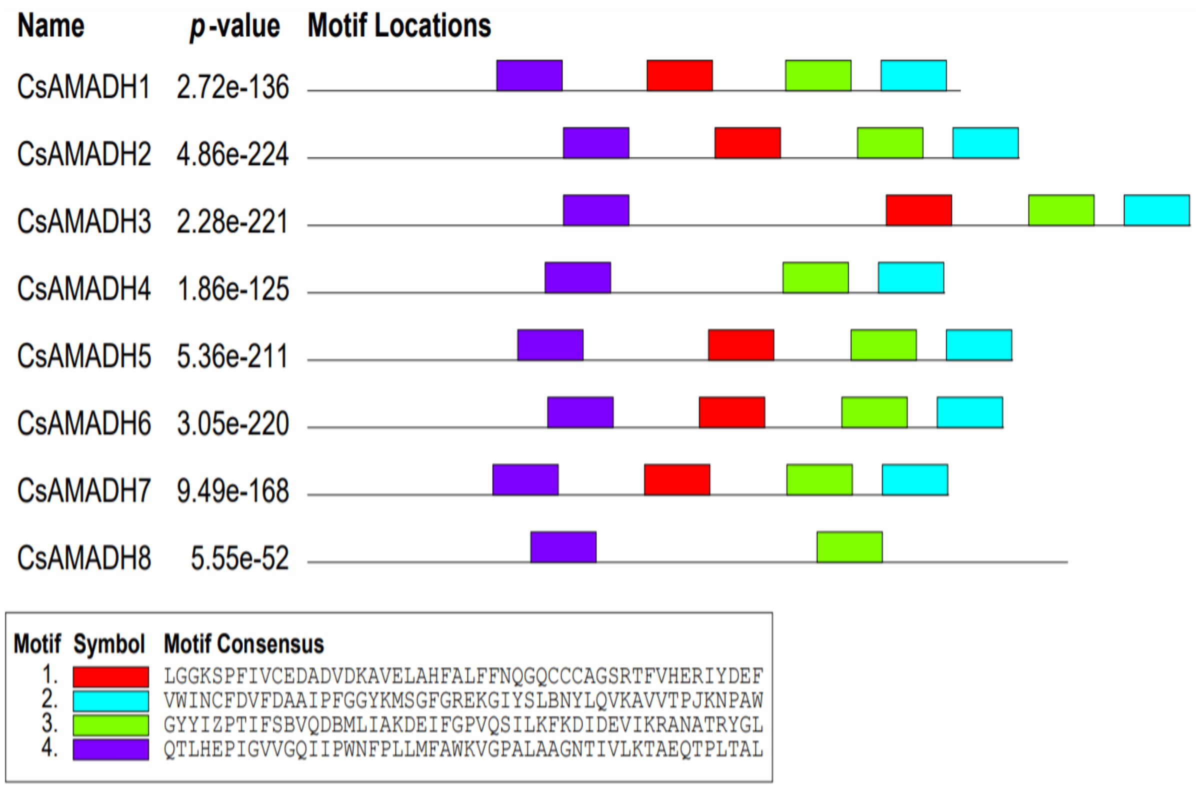

Name
p-value
Motif Locations
CsAMADH1 2.72e-136
CsAMADH2 4.86e-224
CsAMADH3 2.28e-221
CsAMADH4 1.86e-125
CsAMADH5 5.36e-211
CsAMADH6 3.05e-220
CsAMADH7 9.49e-168
CsAMADH8 5.55e-52
Motif Symbol Motif Consensus
1. LGGKSPFIVCEDADVDKAVELAHFALFFNQGQCCCAGSRTFVHERIYDEF
2. VWINCFDVFDAAIPFGGYKMSGFGREKGIYSLBNYLQVKAVVTPJKNPAW
3. GYYIZPTIFSBVQDBMLIAKDEIFGPVQSILKFKDIDEVIKRANATRYGL
4. QTLHEPIGVVGQIIPWNFPLLMFAWKVGPALAAGNTIVLKTAEQTPLTAL

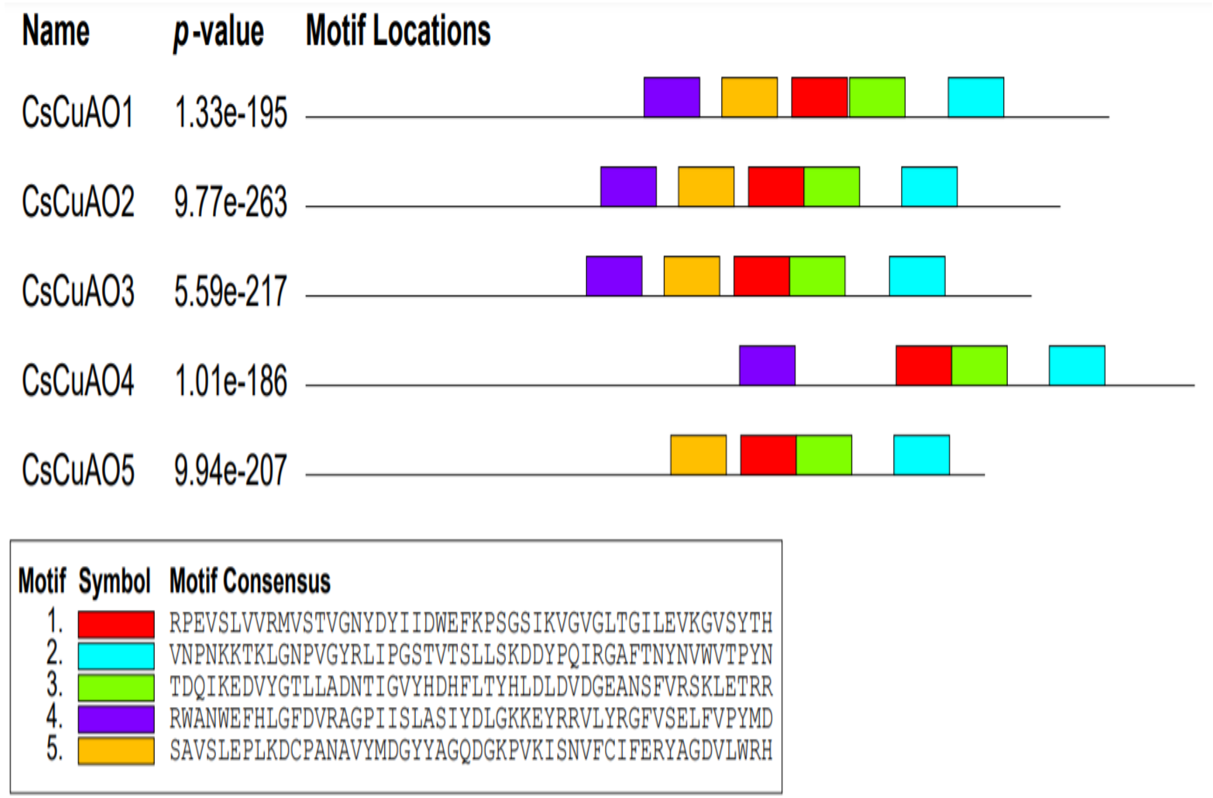

Name
p-value
Motif Locations
CsCuAO1 1.33e-195
CsCuAO2 9.77e-263
CsCuAO3 5.59e-217
CsCuAO4 1.01e-186
CsCuAO5 9.94e-207
Motif Symbol Motif Consensus
1. RPEVSLVVRMVSTVGNYDYIIDWEFKPSGSIKVGVGLTGILEVKGVSYTH
2. VNPNKKTKLGNPVGYRLIPGSTVTSLLSKDDYPQIRGAFTNYNVWVTPYN
3. TDQIKEDVYGTLLADNTIGVYHDHFLTYHLDLDVDGEANSFVRSKLETRR
4. RWANWEFHLGFDVRAGPIISLASIYDLGKKEYRRVLYRGFVSELFVPYMD
5. SAVSLEPLKDCPANAVYMDGYYAGQDGKPVKISNVFCIFERYAGDVLWRH

Figure S1

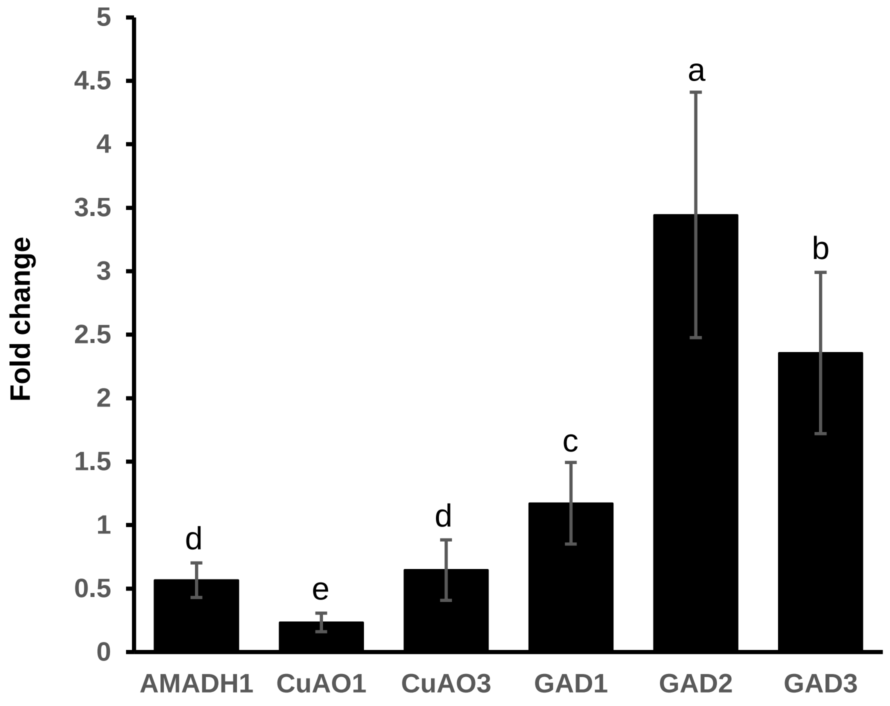

Fold change
5
4.5
4
3.5
3
2.5
2
1.5
1
0.5
0
a
b
c
d
d
e
AMADH1
CuAO1
CuAO3
GAD1
GAD2
GAD3

Figure S2
